# *N*-(4-Cyano­phen­yl)-2,6-difluoro­benzamide

**DOI:** 10.1107/S1600536810046507

**Published:** 2010-11-17

**Authors:** Hoong-Kun Fun, Jia Hao Goh, Janardhana Gowda, A. M. Khader, B. Kalluraya

**Affiliations:** a X-ray Crystallography Unit, School of Physics, Universiti Sains Malaysia, 11800 USM, Penang, Malaysia; b Department of Studies in Chemistry, Mangalore University, Mangalagangotri, Mangalore 574 199, India

## Abstract

In the title compound, C_14_H_8_F_2_N_2_O, the amide plane is inclined at dihedral angles of 28.12 (12) and 32.89 (12)° with respect to the two benzene rings; the dihedral angle between the two rings is 5.58 (5)°. In the crystal, inter­molecular N—H⋯O and C—H⋯F hydrogen bonds link adjacent mol­ecules into a double-chain structure along the *b* axis.

## Related literature

For general background to and applications of the title compound, see: Ashwood *et al.* (1990 ▶); Kees *et al.* (1989 ▶); Ragavan *et al.* (2010 ▶); Carmellino *et al.* (1994 ▶); Rauko *et al.* (2001 ▶). For a closely related benzamide structure, see: Cronin *et al.* (2000 ▶). For the stability of the temperature controller used in the data collection, see: Cosier & Glazer (1986 ▶).
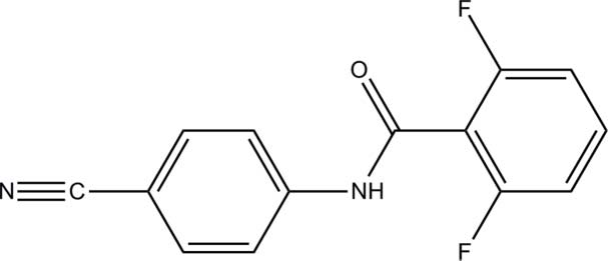


## Experimental

### 

#### Crystal data

C_14_H_8_F_2_N_2_O
*M* *_r_* = 258.22
Monoclinic, 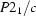

*a* = 9.3377 (11) Å
*b* = 5.0793 (6) Å
*c* = 24.500 (3) Å
β = 100.202 (3)°
*V* = 1143.6 (2) Å^3^
*Z* = 4
Mo *K*α radiation
μ = 0.12 mm^−1^
*T* = 100 K
0.27 × 0.14 × 0.14 mm

#### Data collection

Bruker APEXII DUO CCD area-detector diffractometer
Absorption correction: multi-scan (*SADABS*; Bruker, 2009 ▶) *T*_min_ = 0.968, *T*_max_ = 0.984
27219 measured reflections
4135 independent reflections
3176 reflections with *I* > 2σ(*I*)
*R*_int_ = 0.054

#### Refinement

*R*[*F* ^2^ > 2σ(*F* ^2^)] = 0.043
*wR*(*F* ^2^) = 0.118
*S* = 1.03
4135 reflections
204 parameters
All H-atom parameters refined
Δρ_max_ = 0.39 e Å^−3^
Δρ_min_ = −0.23 e Å^−3^

### 

Data collection: *APEX2* (Bruker, 2009 ▶); cell refinement: *SAINT* (Bruker, 2009 ▶); data reduction: *SAINT*; program(s) used to solve structure: *SHELXTL* (Sheldrick, 2008 ▶); program(s) used to refine structure: *SHELXTL*; molecular graphics: *SHELXTL*; software used to prepare material for publication: *SHELXTL* and *PLATON* (Spek, 2009 ▶).

## Supplementary Material

Crystal structure: contains datablocks global, I. DOI: 10.1107/S1600536810046507/is2630sup1.cif

Structure factors: contains datablocks I. DOI: 10.1107/S1600536810046507/is2630Isup2.hkl

Additional supplementary materials:  crystallographic information; 3D view; checkCIF report

## Figures and Tables

**Table 1:** Hydrogen-bond geometry (Å, °)

*D*—H⋯*A*	*D*—H	H⋯*A*	*D*⋯*A*	*D*—H⋯*A*
N1—H1*N*1⋯O1^i^	0.863 (15)	2.107 (15)	2.9029 (12)	153.3 (14)
C12—H12⋯F1^ii^	0.940 (16)	2.473 (16)	3.4066 (14)	172.4 (13)

Symmetry codes: (i) 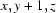; (ii) 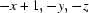.

**Fig. 1.**
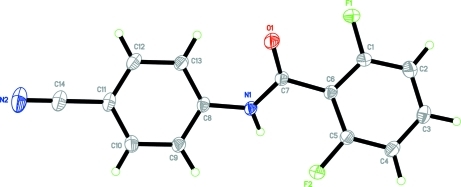
The molecular structure of the title compound, showing 50 % probability displacement ellipsoids for non-H atoms and the atom-numbering scheme.

**Fig. 2.**
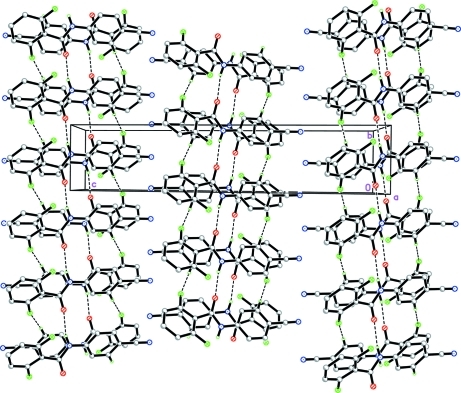
The crystal structure of the title compound, viewed along the a axis, showing two-molecule-wide infinite chains along the [010] direction. H atoms not involved in intermolecular hydrogen bonds (dashed lines) have been omitted for clarity.

## supplementary crystallographic information

### Comment

A number of benzamide derivatives were reported as anti-hypertensive (Ashwood *et al.*, 1990), anti-diabetic (Kees *et al.*, 1989), anti-bacterial (Ragavan *et al.*, 2010), anti-fungal (Carmellino *et al.*, 1994) and anti-cancer (Rauko *et al.*, 2001) activities. On the basis of these considerations, our particular attention was paid for the synthesis of some benzamide derivatives.

In the title benzamide derivative, the amino moiety (C7/N1/O1) is essentially planar, as indicated by the C7–O1–N1–H1N1 torsion angle of -1.4 (18)°. The mean plane through the amido moiety is inclined at dihedral angles of 32.89 (12) and 28.12 (12)°, respectively, with the C1–C6 and C8–C13 benzene rings. The dihedral angle between the two benzene rings being 5.58 (5)°. All bond lengths and angles are comparable to values observed in a closely related benzamide structure (Cronin *et al.*, 2000). In the crystal packing, adjacent molecules are interconnected into two-molecule-wide infinite chains propagating along the [010] direction (Fig. 2) *via* intermolecular N1—H1N1···O1 and C12—H12···F1 hydrogen bonds (Table 1).

### Experimental

A mixture of 4-amino benzonitrile (4.2 mmol), 2,6-difluorobenzoic acid (4.6 mmol) and triethyl amine (21 mmol) was dissolved in methylene dichloride (5 ml). The resulting solution was cooled to 273 K followed by the drop wise addition of 50 % phosphoric acid cyclic anhydride solution in ethyl acetate (4 ml, 6.3 mmol) and stirred for 12 h. The completion of reaction was checked by TLC. Evaporation of solvent gave 2,6-difluoro-*N*-(*p*-cyanophenyl)benzamide as solid mass, which was then stirred with saturated NaHCO_3_ solution to remove excess of acid. Single crystals suitable for X-ray analysis were obtained by crystallization from acetone under slow evaporation. *M.p.* 418 K.

### Refinement

All H atoms were located from a difference Fourier map and allowed to refine freely [refined distances: N—H = 0.860 (16) Å and C—H = 0.933 (15)–0.984 (15) Å].

### Crystal data

**Table:**

C_14_H_8_F_2_N_2_O	*F*(000) = 528
*M**_r_* = 258.22	*D*_x_ = 1.500 Mg m^−^^3^
Monoclinic, *P*2_1_/*c*	Mo *K*α radiation, λ = 0.71073 Å
Hall symbol: -P 2ybc	Cell parameters from 5020 reflections
*a* = 9.3377 (11) Å	θ = 2.2–31.3°
*b* = 5.0793 (6) Å	µ = 0.12 mm^−^^1^
*c* = 24.500 (3) Å	*T* = 100 K
β = 100.202 (3)°	Block, yellow
*V* = 1143.6 (2) Å^3^	0.27 × 0.14 × 0.14 mm
*Z* = 4	

### Data collection

**Table:**

Bruker APEXII DUO CCD area-detector diffractometer	4135 independent reflections
Radiation source: fine-focus sealed tube	3176 reflections with *I* > 2σ(*I*)
graphite	*R*_int_ = 0.054
φ and ω scans	θ_max_ = 32.6°, θ_min_ = 2.2°
Absorption correction: multi-scan (*SADABS*; Bruker, 2009)	*h* = −14→14
*T*_min_ = 0.968, *T*_max_ = 0.984	*k* = −7→7
27219 measured reflections	*l* = −36→37

### Refinement

**Table:**

Refinement on *F*^2^	Primary atom site location: structure-invariant direct methods
Least-squares matrix: full	Secondary atom site location: difference Fourier map
*R*[*F*^2^ > 2σ(*F*^2^)] = 0.043	Hydrogen site location: inferred from neighbouring sites
*wR*(*F*^2^) = 0.118	All H-atom parameters refined
*S* = 1.03	*w* = 1/[σ^2^(*F*_o_^2^) + (0.0568*P*)^2^ + 0.2429*P*] where *P* = (*F*_o_^2^ + 2*F*_c_^2^)/3
4135 reflections	(Δ/σ)_max_ < 0.001
204 parameters	Δρ_max_ = 0.39 e Å^−^^3^
0 restraints	Δρ_min_ = −0.23 e Å^−^^3^

### Special details

**Table:**

Experimental. The crystal was placed in the cold stream of an Oxford Cryosystems Cobra open-flow nitrogen cryostat (Cosier & Glazer, 1986) operating at 100.0 (1)K.
Geometry. All esds (except the esd in the dihedral angle between two l.s. planes) are estimated using the full covariance matrix. The cell esds are taken into account individually in the estimation of esds in distances, angles and torsion angles; correlations between esds in cell parameters are only used when they are defined by crystal symmetry. An approximate (isotropic) treatment of cell esds is used for estimating esds involving l.s. planes.
Refinement. Refinement of F^2^ against ALL reflections. The weighted R-factor wR and goodness of fit S are based on F^2^, conventional R-factors R are based on F, with F set to zero for negative F^2^. The threshold expression of F^2^ > 2sigma(F^2^) is used only for calculating R-factors(gt) etc. and is not relevant to the choice of reflections for refinement. R-factors based on F^2^ are statistically about twice as large as those based on F, and R- factors based on ALL data will be even larger.

### Fractional atomic coordinates and isotropic or equivalent isotropic displacement parameters (Å^2^)

**Table:**

	*x*	*y*	*z*	*U*_iso_*/*U*_eq_	
F1	0.81277 (8)	0.06726 (14)	0.14252 (3)	0.03127 (18)	
F2	1.02229 (7)	0.75952 (13)	0.05423 (3)	0.02670 (16)	
O1	0.76102 (9)	0.12482 (15)	0.03141 (3)	0.02539 (18)	
N1	0.75319 (9)	0.56187 (17)	0.01066 (3)	0.01774 (17)	
N2	0.33842 (13)	0.5436 (2)	−0.24586 (5)	0.0384 (3)	
C1	0.91364 (12)	0.2568 (2)	0.14216 (4)	0.0207 (2)	
C2	1.01589 (13)	0.2889 (2)	0.18972 (5)	0.0252 (2)	
C3	1.11850 (12)	0.4873 (2)	0.19138 (5)	0.0246 (2)	
C4	1.11804 (11)	0.6494 (2)	0.14589 (5)	0.0228 (2)	
C5	1.01538 (11)	0.6060 (2)	0.09898 (4)	0.01910 (19)	
C6	0.90844 (10)	0.41080 (19)	0.09449 (4)	0.01688 (18)	
C7	0.80070 (11)	0.35107 (19)	0.04285 (4)	0.01768 (19)	
C8	0.66236 (11)	0.55108 (19)	−0.04175 (4)	0.01768 (19)	
C9	0.67895 (12)	0.7489 (2)	−0.07969 (4)	0.0207 (2)	
C10	0.59456 (12)	0.7492 (2)	−0.13217 (5)	0.0224 (2)	
C11	0.49135 (11)	0.5511 (2)	−0.14682 (4)	0.0220 (2)	
C12	0.47172 (12)	0.3569 (2)	−0.10873 (5)	0.0231 (2)	
C13	0.55706 (11)	0.3555 (2)	−0.05623 (4)	0.0209 (2)	
C14	0.40564 (13)	0.5474 (2)	−0.20187 (5)	0.0275 (2)	
H1N1	0.7848 (16)	0.716 (3)	0.0216 (6)	0.031 (4)*	
H2	1.0120 (16)	0.173 (3)	0.2194 (6)	0.031 (4)*	
H3	1.1931 (16)	0.507 (3)	0.2249 (6)	0.031 (4)*	
H4	1.1877 (17)	0.787 (3)	0.1456 (6)	0.028 (4)*	
H9	0.7526 (15)	0.877 (3)	−0.0683 (6)	0.022 (3)*	
H10	0.6115 (15)	0.879 (3)	−0.1573 (6)	0.026 (4)*	
H12	0.3999 (17)	0.227 (3)	−0.1180 (6)	0.031 (4)*	
H13	0.5408 (15)	0.221 (3)	−0.0309 (6)	0.026 (4)*	

### Atomic displacement parameters (Å^2^)

**Table:**

	*U*^11^	*U*^22^	*U*^33^	*U*^12^	*U*^13^	*U*^23^
F1	0.0336 (4)	0.0283 (4)	0.0293 (4)	−0.0134 (3)	−0.0015 (3)	0.0072 (3)
F2	0.0242 (3)	0.0266 (3)	0.0274 (3)	−0.0063 (3)	−0.0006 (3)	0.0092 (3)
O1	0.0327 (4)	0.0133 (3)	0.0260 (4)	−0.0007 (3)	−0.0061 (3)	−0.0027 (3)
N1	0.0212 (4)	0.0123 (4)	0.0175 (4)	−0.0004 (3)	−0.0026 (3)	−0.0018 (3)
N2	0.0415 (6)	0.0392 (6)	0.0283 (5)	0.0086 (5)	−0.0108 (5)	−0.0071 (5)
C1	0.0227 (5)	0.0172 (5)	0.0215 (5)	−0.0029 (4)	0.0021 (4)	0.0004 (3)
C2	0.0283 (5)	0.0265 (5)	0.0195 (5)	−0.0012 (4)	0.0011 (4)	0.0017 (4)
C3	0.0235 (5)	0.0285 (6)	0.0198 (5)	−0.0004 (4)	−0.0018 (4)	−0.0042 (4)
C4	0.0190 (5)	0.0222 (5)	0.0257 (5)	−0.0025 (4)	−0.0003 (4)	−0.0029 (4)
C5	0.0187 (4)	0.0174 (4)	0.0206 (4)	0.0002 (3)	0.0020 (3)	0.0004 (3)
C6	0.0173 (4)	0.0145 (4)	0.0179 (4)	0.0004 (3)	0.0007 (3)	−0.0023 (3)
C7	0.0188 (4)	0.0144 (4)	0.0187 (4)	0.0009 (3)	0.0002 (3)	−0.0014 (3)
C8	0.0186 (4)	0.0156 (4)	0.0176 (4)	0.0020 (3)	−0.0003 (3)	−0.0030 (3)
C9	0.0240 (5)	0.0170 (4)	0.0194 (5)	−0.0008 (4)	−0.0007 (4)	−0.0015 (3)
C10	0.0264 (5)	0.0202 (5)	0.0189 (5)	0.0021 (4)	−0.0002 (4)	−0.0006 (4)
C11	0.0214 (5)	0.0226 (5)	0.0195 (5)	0.0049 (4)	−0.0037 (4)	−0.0055 (4)
C12	0.0196 (5)	0.0206 (5)	0.0266 (5)	−0.0003 (4)	−0.0030 (4)	−0.0051 (4)
C13	0.0206 (5)	0.0181 (5)	0.0224 (5)	−0.0004 (4)	−0.0008 (4)	−0.0012 (4)
C14	0.0279 (5)	0.0259 (5)	0.0254 (5)	0.0056 (4)	−0.0043 (4)	−0.0057 (4)

### Geometric parameters (Å, °)

**Table:**

F1—C1	1.3480 (12)	C4—H4	0.956 (15)
F2—C5	1.3563 (12)	C5—C6	1.3975 (14)
O1—C7	1.2246 (12)	C6—C7	1.5014 (13)
N1—C7	1.3569 (13)	C8—C9	1.3961 (14)
N1—C8	1.4091 (12)	C8—C13	1.3985 (14)
N1—H1N1	0.860 (16)	C9—C10	1.3839 (15)
N2—C14	1.1474 (15)	C9—H9	0.953 (14)
C1—C2	1.3791 (15)	C10—C11	1.3955 (15)
C1—C6	1.3992 (14)	C10—H10	0.933 (15)
C2—C3	1.3861 (16)	C11—C12	1.3922 (16)
C2—H2	0.943 (15)	C11—C14	1.4414 (15)
C3—C4	1.3849 (16)	C12—C13	1.3880 (15)
C3—H3	0.984 (15)	C12—H12	0.939 (15)
C4—C5	1.3777 (14)	C13—H13	0.952 (15)
			
C7—N1—C8	125.52 (8)	O1—C7—C6	120.86 (9)
C7—N1—H1N1	118.5 (10)	N1—C7—C6	115.57 (8)
C8—N1—H1N1	115.9 (10)	C9—C8—C13	119.89 (9)
F1—C1—C2	117.31 (9)	C9—C8—N1	117.32 (9)
F1—C1—C6	118.94 (9)	C13—C8—N1	122.78 (9)
C2—C1—C6	123.75 (10)	C10—C9—C8	120.48 (10)
C1—C2—C3	118.83 (10)	C10—C9—H9	122.3 (8)
C1—C2—H2	117.6 (9)	C8—C9—H9	117.2 (8)
C3—C2—H2	123.6 (9)	C9—C10—C11	119.47 (10)
C4—C3—C2	120.33 (10)	C9—C10—H10	118.6 (9)
C4—C3—H3	120.8 (9)	C11—C10—H10	121.9 (9)
C2—C3—H3	118.8 (9)	C12—C11—C10	120.36 (9)
C5—C4—C3	118.60 (10)	C12—C11—C14	120.06 (10)
C5—C4—H4	119.0 (9)	C10—C11—C14	119.58 (10)
C3—C4—H4	122.3 (9)	C13—C12—C11	120.18 (10)
F2—C5—C4	117.17 (9)	C13—C12—H12	119.3 (9)
F2—C5—C6	118.68 (9)	C11—C12—H12	120.5 (9)
C4—C5—C6	124.13 (10)	C12—C13—C8	119.60 (10)
C5—C6—C1	114.34 (9)	C12—C13—H13	118.5 (9)
C5—C6—C7	124.92 (9)	C8—C13—H13	121.9 (9)
C1—C6—C7	120.62 (9)	N2—C14—C11	179.41 (13)
O1—C7—N1	123.57 (9)		
			
F1—C1—C2—C3	−178.04 (10)	C1—C6—C7—O1	−31.41 (15)
C6—C1—C2—C3	1.20 (18)	C5—C6—C7—N1	−35.04 (14)
C1—C2—C3—C4	−0.17 (18)	C1—C6—C7—N1	149.05 (10)
C2—C3—C4—C5	−1.07 (17)	C7—N1—C8—C9	−149.79 (11)
C3—C4—C5—F2	−176.83 (10)	C7—N1—C8—C13	31.27 (16)
C3—C4—C5—C6	1.43 (17)	C13—C8—C9—C10	−1.75 (16)
F2—C5—C6—C1	177.77 (9)	N1—C8—C9—C10	179.28 (10)
C4—C5—C6—C1	−0.47 (15)	C8—C9—C10—C11	0.62 (16)
F2—C5—C6—C7	1.63 (15)	C9—C10—C11—C12	0.98 (16)
C4—C5—C6—C7	−176.61 (10)	C9—C10—C11—C14	−178.58 (10)
F1—C1—C6—C5	178.35 (9)	C10—C11—C12—C13	−1.44 (16)
C2—C1—C6—C5	−0.88 (16)	C14—C11—C12—C13	178.11 (10)
F1—C1—C6—C7	−5.33 (15)	C11—C12—C13—C8	0.31 (16)
C2—C1—C6—C7	175.44 (10)	C9—C8—C13—C12	1.28 (16)
C8—N1—C7—O1	−5.34 (17)	N1—C8—C13—C12	−179.81 (10)
C8—N1—C7—C6	174.18 (9)	C7—O1—N1—H1N1	−1.4 (18)
C5—C6—C7—O1	144.50 (11)		

### Hydrogen-bond geometry (Å, °)

**Table:**

*D*—H···*A*	*D*—H	H···*A*	*D*···*A*	*D*—H···*A*
N1—H1N1···O1^i^	0.863 (15)	2.107 (15)	2.9029 (12)	153.3 (14)
C12—H12···F1^ii^	0.940 (16)	2.473 (16)	3.4066 (14)	172.4 (13)

Symmetry codes: (i) *x*, *y*+1, *z*; (ii) −*x*+1, −*y*, −*z*.

## References

[bb1] Ashwood, V. A., Cassidy, F., Coldwell, M. C., Evans, J. M., Hamilton, T. C., Howlett, D. R., Smith, D. M. & Stemp, G. (1990). *J. Med. Chem.* **33**, 2667–2672. 10.1021/jm00171a051 2391705

[bb2] Bruker (2009). *APEX2*, *SAINT* and *SADABS* Bruker AXS Inc., Madison, Wisconsin, USA.

[bb3] Carmellino, M. L., Pagani, G., Pregnolato, M., Terreni, M. & Pastoni, F. (1994). *Eur. J. Med. Chem.* **29**, 743–751.

[bb4] Cosier, J. & Glazer, A. M. (1986). *J. Appl. Cryst.* **19**, 105–107.

[bb5] Cronin, L., Adams, D. A., Nightingale, D. J. & Clark, J. H. (2000). *Acta Cryst.* C**56**, 244–245. 10.1107/s0108270199014651 10777904

[bb6] Kees, K. L., Cheeseman, R. S., Prozialeck, D. H. & Steiner, K. E. (1989). *J. Med. Chem.* **32**, 11–13. 10.1021/jm00121a003 2642552

[bb7] Ragavan, R. V., Vijayakumar, V. & Suchetha Kumari, N. (2010). *Eur. J. Med. Chem.* **43**, 1173–1180. 10.1016/j.ejmech.2009.12.042 20053480

[bb8] Rauko, P., Novotny, L., Dovinova, I., Hunakova, L., Szekeres, T. & Jayaram, H. N. (2001). *Eur. J. Pharm. Sci.* **12**, 387–394. 10.1016/s0928-0987(00)00180-9 11231105

[bb9] Sheldrick, G. M. (2008). *Acta Cryst.* A**64**, 112–122. 10.1107/S0108767307043930 18156677

[bb10] Spek, A. L. (2009). *Acta Cryst.* D**65**, 148–155. 10.1107/S090744490804362X PMC2631630 19171970

